# Reactive Oxygen Species Induced Pathways in Heart Failure Pathogenesis and Potential Therapeutic Strategies

**DOI:** 10.3390/biomedicines10030602

**Published:** 2022-03-03

**Authors:** Aušra Mongirdienė, Laurynas Skrodenis, Leila Varoneckaitė, Gerda Mierkytė, Justinas Gerulis

**Affiliations:** 1 Department of Biochemistry, Medical Academy, Lithuanian University of Health Sciences, Eiveniu str. 4, LT-50161 Kaunas, Lithuania; 2 Medical Academy, Lithuanian University of Health Sciences, Mickevičiaus str. 9, LT-44307 Kaunas, Lithuania; laurynas.skrodenis@stud.lsmu.lt (L.S.); leila.varoneckaite@stud.lsmu.lt (L.V.); gerda.mierkyte@stud.lsmu.lt (G.M.); justinas.gerulis@stud.lsmu.lt (J.G.)

**Keywords:** heart failure with reduced ejection fraction, heart failure with preserved ejection fraction, reactive oxygen species, protein kinases, NO, cGC

## Abstract

With respect to structural and functional cardiac disorders, heart failure (HF) is divided into HF with reduced ejection fraction (HFrEF) and HF with preserved ejection fraction (HFpEF). Oxidative stress contributes to the development of both HFrEF and HFpEF. Identification of a broad spectrum of reactive oxygen species (ROS)-induced pathways in preclinical models has provided new insights about the importance of ROS in HFrEF and HFpEF development. While current treatment strategies mostly concern neuroendocrine inhibition, recent data on ROS-induced metabolic pathways in cardiomyocytes may offer additional treatment strategies and targets for both of the HF forms. The purpose of this article is to summarize the results achieved in the fields of: (1) ROS importance in HFrEF and HFpEF pathophysiology, and (2) treatments for inhibiting ROS-induced pathways in HFrEF and HFpEF patients. ROS-producing pathways in cardiomyocytes, ROS-activated pathways in different HF forms, and treatment options to inhibit their action are also discussed.

## 1. Introduction

With respect to structural and functional cardiac disorders, chronic heart failure (CHF) is classified into heart failure (HF) with reduced ejection fraction (HFrEF) and HF with preserved ejection fraction (HFpEF). The most common cause of HFrEF is cardiomyocyte loss due to ischemia. HFpEF is a heterogeneous syndrome with multiple different conditions that can contribute differently to the syndrome [1]. Patients with HFpEF make up more than 50% of all HF patients [1,2]. In recent decades, understanding of the pathophysiology and treatment of HFrEF has increased [3,4]. However, treatment options for patients with HFpEF are few [1]. The main therapeutic target for patients with HFrEF is the neuroendocrine chain with therapies including inhibitors of the renin angiotensin-aldosterone system, mineralocorticoid receptor antagonists, β-receptor blockers, and medications that increase the half-life of natriuretic peptides [5]. HFpEF treatment relies on addressing the reasons for the observed syndrome, including treating the underlying disease, blood pressure control, use of diuretics and addressing other factors that contribute to development of HFpEF [1].

Oxidative stress (an imbalance between the increased formation of reactive oxygen species (ROS) and the elimination or neutralization of ROS by an antioxidant system) plays an important role in the development of CHF [6] and correlates with left ventricle (LV) dysfunction and hypertrophy in the failing heart [7]. Therefore, pharmacologically targeting specific ROS and pathways induced by them, could be beneficial for CHF patients. Broad and detailed knowledge of the particular sources and formation of ROS, as well as their elimination in the cell, is required in order to better understand the ROS induced pathways. Therefore, we have sought to summarize results achieved in the fields of: (1) ROS significance for HFrEF and HFpEF pathophysiology, and (2) treatment options in the management of ROS-induced pathways in the human heart for both HFrEF and HFpEF patients.

## 2. ROS Sources, Importance and Danger in Human Cells

**Figure 1:**
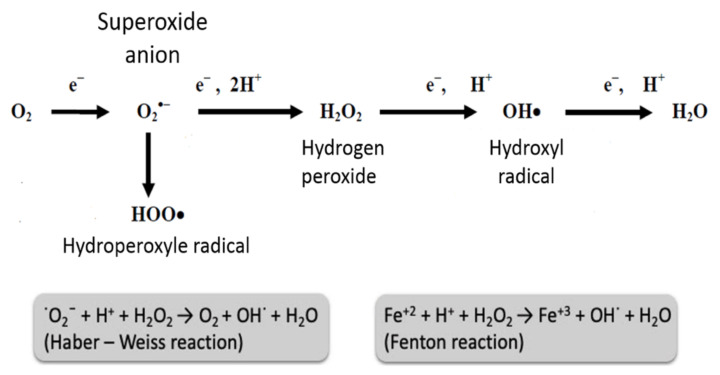
Reactive oxygen species. O_2_^•−^-superoxide anion, OH^•^-hydroxyl radical, H_2_O_2_-hydrogen peroxide, HOO^•^-hydroperoxyle radical, H_2_O-water.

ROS are chemically reactive molecules that belong to a group of nine major types of free radicals. These molecules have an unpaired electron in the superoxide anion O_2_^−^, which is unstable. Several compounds are termed ROS, including: free radicals (superoxide anion (O_2_^−^)), the hydroxyl radical (^.^OH)) and oxidative agents (e.g., hydrogen peroxide (H_2_O_2_), peroxynitrite (ONOO^−^), hypochlorite (OCl^−^)) [8,9]. H_2_O_2_ is involved in the Fenton reaction in the presence of Fe^2+^ to produce –OH. O_2_^−^ and H_2_O_2_ can produce –OH through the Haber–Weiss reaction [10]. ONOO^−^ is produced in the reaction of ^.^NO with O_2_^−^ [11] (Figure 1) and is known to contribute to chronic heart failure (CHF) pathogenesis [12].

**Figure 2:**
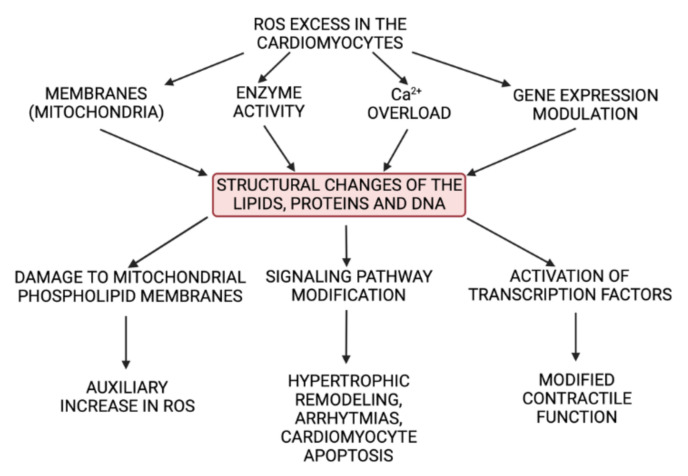
Harmful effects of ROS in cardiomyocytes (created with BioRender.com on 11 February 2022).

The agents referred to are produced in the cells by the mitochondria and enzymes, such as lipoxygenases and cyclooxygenases, under normal conditions [13]. Some processes, such as apoptosis, immune system reactions, differentiation, activation of several transcriptional factors, cellular signaling pathways and induction of a mitogenic response require the presence of some ROS [14]. ROS signaling is either reversible and oxidative or produces reactive nitrogen species. O^2•−^ takes part in signal transmission by: (1) causing post-translation redox modifications of proteins [15], (2) hydroxylation (addition of an HO group) [16], and (3) S-nitrosylation (oxidation of cysteine by NO) [17]. By these means, the reactivity, stability and conformation of the affected molecules is altered [17]. The superoxide anion and hydrogen peroxide are the main ROS that participate in redox signaling [18] (Figure 1). Hydroxyl radicals are more reactive and less specific and reversible [19]. Cell antioxidant enzymes, including superoxide dismutase, catalase, and glutathione peroxidase, protect the cell from ROS excess [20]. ROS excess is known to give rise to oxidative stress, which affects subcellular organelles, changes intracellular enzyme activity, creates intracellular Ca^2+^ overload and modulates gene expression [21,22]. In turn, cell lipids, proteins and deoxyribonucleic acid (DNA) are damaged, leading to impairment of normal cell function [22] (Figure 2).

ROS, as reported in the literature, activate the signal kinase and transcription factors that modify the function of intracellular proteins and signaling pathways in the heart and in this way contribute to the hypertrophic remodeling of the heart [23,24]. Additionally, ROS damage mitochondrial phospholipid membranes and, as a consequence, induce mitochondrial oxidative stress, leading to molecular mechanisms that contribute to the development and progression of heart failure [25].

## 3. ROS in the Pathogenesis of CHF Development

Being a by-product of aerobic metabolism, ROS are abundant in the cells of the myocardium and, if the balance between ROS production and antioxidant systems is impaired, they can greatly contribute to, or worsen, HF [26].

The proteins involved in redox signaling are protein kinase G (PKG) [27], the small G protein Ras [28], Ca/calmodulin-dependent protein kinase II (CaMKII) [29], protein kinase A (PKA) [30], class II histone deacetylases (HDACs) [31], matrix metalloproteinase (MMP) [32], protein kinase B/Akt [33], the extracellular signal-regulated kinase ½ (ERK1/2) [34], p38 MAP kinase [35], protein kinase C (PKC) [36], NF-kappa B [37], and transcription factors, including activated protein-1 [38].

Cardiomyocyte hypertrophy has been found to be associated with ROS activation of signaling kinases and transcription factors [23]. ROS also promotes post-translational modifications that change the function of specific proteins and signaling pathways, leading to hypertrophic remodeling [23,39]. ROS have been shown to be important in G protein-coupled receptor stimulation by angiotensin II, tumor necrosis factor-α (TNF-α), and α-adrenergic stimulation [23,40,41]. Angiotensin II may participate in myocardial hypertrophy by several intracellular pathways by activating: (1) protein kinase C, (2) c-Jun N-terminal kinase (JNK), (3) extracellular signal-regulated kinase, and (4) ROS formation [40,42]. Though the role of TNF-α in cardiomyocytes is not yet sufficiently known, TNF-α seems to play an autocrine or paracrine role in activating MMPs, which promote hypertrophic changes in the heart [41].

ROS affect different lipid membranes too, including the sarcolemma, mitochondrial membranes, nuclear membrane, and the sarcoplasmic reticulum, in which lipid radicals and lipid hydroperoxide (LPH) form [25,43,44]. As the lipid peroxidation cascade progresses, LPH reacts with fatty acids to form a more stable product, for example—malondialdehyde or 4-hydroxy-2-nonenal [45]. Destabilization of the phospholipid-rich inner mitochondrial membrane by peroxidation results in additional electron leakage and increase in ROS production intensity [43,45].

ROS activate the cardiac Na^+^/Ca^2+^ exchanger, which triggers cardiac hypertrophy through the Ca^2+^-dependent pathway [46] and contributes to Ca^2+^/calmodulin-dependent protein kinase II activation, leading to increase in cardiomyocyte death and CHF development [47]. The cardiac Na^+^/H^+^ exchanger (NHE1) was shown to be activated [48] and sarcolemmal Na^+^/K^+^ ATPase was found to be suppressed by ROS [49] and to be implicated in cardiac hypertrophy. It should be emphasized that the elevation of Na^+^ in cardiomyocytes may contribute to slower cardiac muscle relaxation and arrhythmias [50].

It should also be mentioned that heme oxygenase (HO) (an enzyme that catalyzes heme degradation) has been shown to reduce oxidative stress in cardiomyocytes by catalyzing the carbon monoxide (CO) producing reaction [50,51]. CO has been shown to act as an antioxidant and contribute to the anti-hypertrophic effect [51].

Additionally, ROS induced endothelial damage [52,53] and thrombosis development [54] are stated in the literature to take place in chronic HF development.

### 3.1. Enzymes Involved in ROS Production

**Figure 3:**
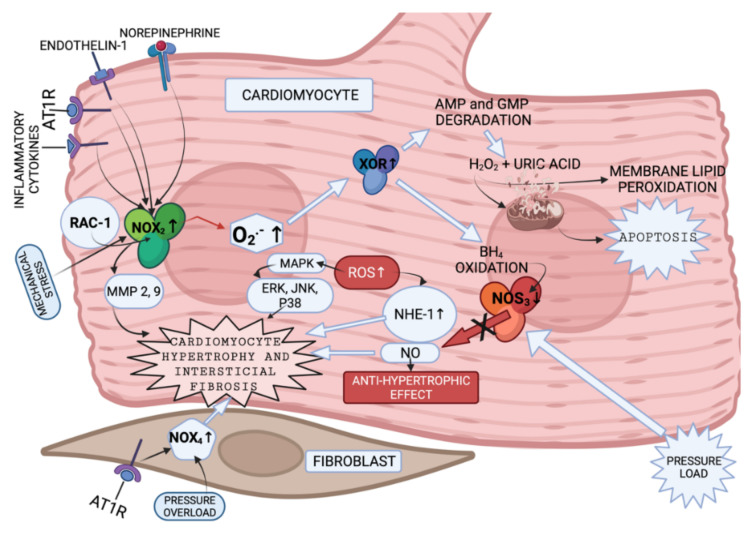
Enzymes involved in ROS production in cardiomyocytes and fibroblasts (created with BioRender.com on 7 February 2022). NOX2 is presented to be activated by endothelin and angiotensin II [58], by cytokines [59] and mechanical stress [60]. Increased NOX2 activation leads to cytoskeletal dysfunction in patients with CHF [61]. It was discovered that superoxide anions, produced by NOX, can oxidize and degrade hydrobiopterin-4 (BH4) leading to NOS uncoupling [62]. Nitric oxide synthase 3 (NOS3) uncoupling was observed in myocardium exposed to chronic pressure load. NOS3 catalyzes nitric oxide (NO) synthesis under physiological conditions. NO has an antihypertrophic effect. However, NOS3 is uncoupled with pressure load, and this, in turn, leads to reduction in tetrahydrobiopterin-4 concentration, increase in ROS production, and, as a consequence, to cardiomyocyte hypertrophy [63]. It was also shown that increase in ROS activates MAPK, leading to increased expression of proteins, such as ERK, JNK and P38, which are related to cardiomyocyte hypertrophy [64] (Figure 3). What is more, NOX-derived ROS may activate XOR [65]. Additionally, angiotensin II-induced signaling and isolated cardiomyocyte hypertrophy are dependent on NOX2 [66]. GTP-binding protein Rac-1 (involved in NOX activation), as described in the literature, is involved in isolated myocyte hypertrophy, induced by endothelin I, phenylephrine, angiotensin II [67] and norepinephrine [68]. AMPK—adenosine monophosphate activated protein kinase; AT1R—angiotensin II receptor; NOX-NADPH oxidase; BH4—dihydrobiopterin-4; NOS—nitric oxide synthase; NO—nitric oxide; MAPK—mitogen-activated protein kinase; XOR—xanthine oxidoreductase; AMP—adenosine monophosphate; GMP—guanosine monophosphate; Rac-1—GTP-binding protein; NHE-1—sodium/hydrogen exchanger-1; ERK—extracellular signal-regulated kinase; JNK-c—Jun N-terminal kinase; p38—a focal point of interactions of the serine/threonine kinases), MMP—matrix metalloproteinase.

NADPH oxidases (NOX) 2 and 4 [55], xanthine oxidoreductase (XOR) [56], and nitric oxide synthase (NOS) [57] are the common enzymes that produce ROS in cardiomyocytes (Figure 3).

Humans have seven NOX with a similar catalytic core, but different regulatory mechanisms [19]. NOX2 and NOX4 are abundantly expressed in cardiomyocytes, endothelium and fibroblasts. Every NOX produces the superoxide anion [62]. NOX2 and NOX4 activity is presented in Figure 3. The data presented in [69,70] implies that NOX4-derived ROS could contribute to overload-induced LV hypertrophy (LVH), and that NOX2 is produced in response to angiotensin II infusion. However, some studies suggest that LVH, as a response to chronic renin-angiotensin-aldosterone system activation, is not associated with NOX2 [71,72]. The enzymes involved in ROS production in cardiomyocytes and fibroblasts, and the pathways that they activate according to the literature [58,59,60,61,62,63,64,65,66,67,68], are presented in summary in Figure 3.

NOX has been shown to be involved in MMP activation in response to angiotensin II [73] and mechanical stretch [71] in the vessels. Experiments with mice and rats have demonstrated the role of NOX2 in the development of interstitial cardiac fibrosis, however, the NOX2-expressing cell type was not established [71,72,74]. NOX4 was shown to be expressed in cardiac fibroblasts [75,76] in animal models. Currently there are no in vivo experiments that could confirm the analogous case in humans.

The other ROS produced enzyme is XOR. XOR is involved in: (1) degradation of the purine nucleotides (AMP and GMP), in which it oxidizes hypoxanthine and xanthine to uric acid and H_2_O_2_ [77], (2) reduction of nitrite and nitrate, in which it produces NO and consequently promotes vasodilatation [78], or inflammation [78] and mitochondrial damage (as a result of overproduction) [79]. There are two forms of XOR: xanthine dehydrogenase and xanthine oxidase (XO). XO is involved in H_2_O_2_ production [80]. High levels of uric acid are found in patients with HF blood tests [77]; therefore, production of H_2_O_2_ is expected to be increased in these patients as well.

NOS catalyze NO production in a reaction where L-arginine is converted to L-citruline [81]. There are three isoforms of NOS. Two isoforms of NOS (endothelial (eNOS) and neuronal (nNOS)) are expressed more intensely in cardiomyocytes [82]. However, iNOS can contribute to contractile damage in CHF as well [83].

Increased ROS production is related both to myosin-activated protein kinase (MAPK) [64] (Figure 3) and adenosine monophosphate activated protein kinase (AMPK) activation [84]. AMPK activation leads to an increase in the antioxidants SOD and catalase (CAT) and uncoupling of protein 2 (UCP2) gene expression, leading to weakened apoptosis and NOX expression decrease [84] (not shown in Figure 3).

Another group of enzymes involved in CHF development through ROS production. is a family of NAD+ dependent class III histone deacetylases called sirtuins (SIRT) [85]. There are seven members of this enzyme group in different cell departments. SIRT3 is found in the mitochondria [86] and is involved in ATP production and ROS detoxication [87,88]. Some studies have found that SIRT3 is involved in cardiac muscle hypertrophy and fibrosis, leading eventually to CHF development [89,90]. Additionally, some studies have found that SIRT3 is involved in oxidative stress-mediated cell death in cardiomyocytes through protein Ku70 deacetylation, leading to deacetylated Ku70 interaction with the apoptosis regulator bcl-2-like protein4 (Bax) [91]. However, other studies have highlighted several mechanisms through which SIRT3 exerts a cardioprotective effect: (1) SIRT3 activates the antioxidant enzyme superoxide dismutase (SOD) [92], and (2) activates isocitrate dehydrogenase 2 (IDH2) by deacetylation. IDH2 uses NADP+ for reduction. SIRT3, in this way, increases NADPH levels, and increases glutathione (GSH) levels, thereby, inhibiting ROS production [93].

It can be concluded that several redox-signaling pathways may be modulated by ROS-producing enzymes, leading to cardiomyocyte hypertrophy, interstitial fibrosis and apoptosis. The value of NOX seems to be far more important in comparison with the other enzymes in that its activation can be triggered by both neuroendocrine factors and pressure overload, or by inflammatory cytokines. NOS play an important role in redox alterations in CHF development, both with substrates and cofactors. Sirtuins are involved in the enzyme-antioxidant activation that protects the heart from hypertrophy. NOX-, XOR-, NOS- and SIRT-mediated pathways could, therefore, be potential treatment targets for CHF development suppression.

### 3.2. Mitochondria in ROS Production and Enzyme-Antioxidants

The most abundant source of ROS in cells is the mitochondrial electron transport chain (ETC). A total of 0.2–2% of the electrons in the ETC leak out of the chain and interact with oxygen to produce superoxide or hydrogen peroxide [47]. Additionally, H_2_O_2_ is produced in the reaction, catalyzed by SOD1. After it has diffused from mitochondria, H_2_O_2_ is involved in physiological and pathological pathways (damaging proteins and lipids) [47]. H_2_O_2_ damages mitochondrial DNA, interferes with the Krebs cycle, ATP production, and fatty acid metabolism [94] and can trigger the opening of ion channels or the inner membrane anion channel inside the mitochondria, leading to cell death [95]. Proton leakage in the mitochondria consists of: (1) basal leakage (not regulated and related to the inner mitochondrial membrane’s lipid bilayer and the adenine nucleotide translocase (ANT)), and (2) inducible leakage (regulated and catalyzed or suppressed by uncoupling proteins and ANT) [47]. UCP2 is involved in cardiovascular disease; therefore, drugs targeting UCP expression or activity might be a potential treatment option. Hypoxia is suggested to further increase ROS production in the mitochondria [47]. ETC complexes III (CIII), and especially I (CI), are found to be the main sites of ROS production [96]. Therefore, the regulation of ROS production in these complexes may yield significant results.

Mitochondrial ROS are also involved in different cell signaling pathways, involving apoptosis [97], autophagy [98], and necrosis [99].

Other ROS sources in the mitochondria are the enzymes monoamine oxidase A and B, both located within the outer mitochondrial membrane (OMM). They catalyze the oxidative deamination of neurotransmitters and biogenic amines, leading to H_2_O_2_ production [100]. Monoamine oxidase A (MAO-A) is specific to cardiomyocytes [101]. It was discovered that MAO-A-dependent ROS formation may impair autophagy, leading to the accumulation of autophagosomes and mitochondrial fusion, resulting in microtubule-associated protein light chain 3-phosphatidylethanolamine conjugate (recruited to autophagosomal membranes) formation, and autophagy receptor (p62) and ubiquitylated protein accumulation, causing cardiomyocyte death and CHF. Both MAO-A derived H_2_O_2_ and aldehydes are able to directly target mitochondrial function [102]. Additionally, MAO-A-generated ROS has been shown to inhibit sphingosine kinase, which leads to ceramide accumulation and, thereby, to cardiomyocyte apoptosis [103]. Contractile proteins (actin and tropomyosin) are also affected [104].

Excess of H_2_O_2_ in the cell is eliminated by glutathione peroxidase (GPX) and peroxiredoxin (PRX). Both of these require GSH and thioredoxin for regeneration [105], which requires NADPH as a cofactor [105,106]. Nicotinamide nucleotide transhydrogenase, NADP+-dependent isocitrate dehydrogenase (IDH) and malic enzyme are involved in NADP+ regeneration to NADPH [107]. These require Krebs cycle products—NADH, malate and isocitrate. With IDH being the most important for NADPH regeneration [108], the Krebs cycle is necessary for the antioxidant capacity within mitochondria [109] as well as in the cytosol [110]. Aldehyde dehydrogenase (ALDH2) is another mitochondrial enzyme involved in antioxidant activity and participates in the detoxication of lipid peroxidation products [111]. Moreover, ROS through intermediate links, activate AMPK, leading to an increase in antioxidant enzyme gene expression (SOD, CAT and (UCP2)) [84].

In sum, the mitochondria are an important ROS source in cardiomyocytes. The amount of ROS produced by mitochondria depends on the supply of oxygen to the cell and activity of the enzymes that produce ROS (especially SOD1 and MAO-A). Cardiomyocyte cytosol antioxidant GPX and PRX regeneration also depends on Krebs cycle action. AMPK activation is important for antioxidant enzyme function.

## 4. Differences in ROS-Induced Pathways between HFrEF and HFpEF

Abnormalities in antioxidant values and oxidative states are found in CHF of various etiologies. Oxidative stress is increased in both HFrEF and HFpEF and is related to the pathogenesis of myocardial remodeling [9,21,55,112,113]. Comparison of oxidative stress readings in ischemic cardiomyopathy and non-ischemic cardiomyopathy patients and the correlations between oxidative stress parameters and clinical readings have highlighted the possibility that the defense mechanisms against ROS could differ between these groups [93].

### 4.1. ROS in HFrEF

HFrEF is considered in patients with an LV ejection fraction lower than 40% [5]. HFrEF is mostly associated with large scale cardiomyocyte death and formation of eccentric hypertrophy as a result of myocardial infarction, cardiomyopathy or valvular heart disease [114]. Irreversible cardiomyopathic changes, subcellular abnormalities and, in turn, decreased heart systolic-diastolic function are suggested to be the causes of elevated ROS levels in HFrEF [115]. Excess of ROS in HFrEF can participate in: (1) subcellular abnormalities and contractile function damage by proteins involved in excitation-contraction and modification coupling [116], (2) myocardial fibroblast proliferation and MMP activation [115], (3) mitochondrial dysfunction due to upsurge of mitochondrial matrix calcium [117], as well as mitochondrial fragmentation, stimulating cardiomyocytes to undergo apoptosis [118] and decrease in oxidative capacity in HFrEF [119]. Lysosomes are known to take part in active mitophagy of clustered mitochondria [117]. Mitophagy is upregulated and intensified with progression to HFrEF [119] due to peroxidation of the lysosomal membranes and lipofuscin accumulation [120].

The molecular mechanisms leading to mitochondrial clustering have not yet been clarified. An mRNA-binding protein, involved in the proper cytoplasmic distribution of mitochondria, named clustered mitochondria protein homolog (Cluh), has been suggested as a participant in mitochondrial biogenesis and oxidative capacity [121]. Cluh has been found to be downregulated in an HFrEF rat model [122], however, it has not yet been investigated in humans.

**Figure 4:**
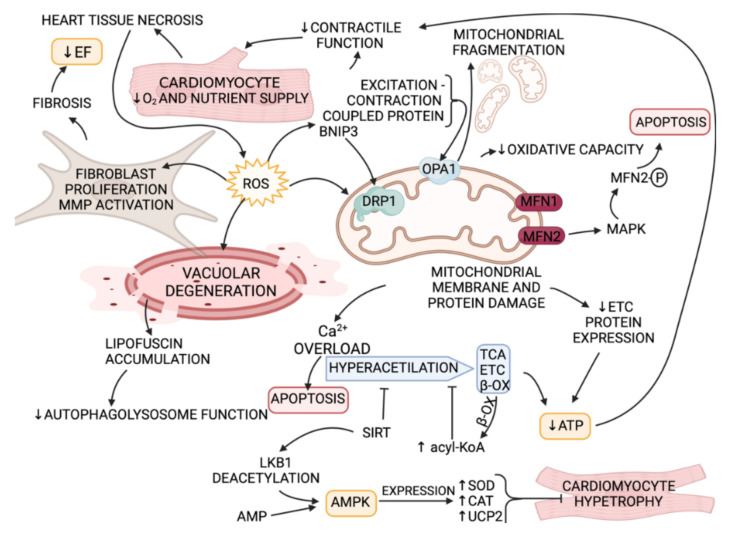
ROS in HFrEF pathogenesis (created with BioRender.com on 9 February 2022). Decrease in O_2_ and nutrient supply in cardiomyocytes results in ROS overproduction. Increase in ROS leads to MMP activation and consequently fibroblast proliferation, vacuolar degeneration, excitation-contraction coupled protein oxidation (consequently leading to decrease in contractile function of cardiomyocytes) and oxidation of mitochondrial OMM and IMM proteins. OMM protein damage results in MAPK pathway activation, leading to apoptosis. IMM protein OPA1 oxidation (resulting in inhibition) with BNIP3 inhibition by ROS causes mitochondrial fragmentation. Both mitochondrial and other cytosol protein oxidation by ROS lead to decrease in ETC protein expression, resulting in ATP production decrease, resulting in poor contraction. Hyperacetylation of ETC complexes, fatty acid beta-oxidation and TCA cycle proteins lead to inhibition of its activity. SIRT, in healthy cardiomyocytes, inhibits hyperacetylation and activates AMPK-induced pathway, leading to enzyme-antioxidant synthesis which leads to hypertrophy inhibition. Excess of ROS inhibits beneficial SIRT effects. (EF—ejection fraction; MMP—matrix metalloproteinase; ROS—reactive oxygen species; BNIP3—mitochondrial mitophagy marker; OPA1—optic atrophy 1 protein; DRP1—dynamin-related protein 1; MFN2—mitofusin 2; MAPK—mitogen-activated protein kinase; ETC—electron transport chain; β-OX—beta-oxidation; SIRT—sirtuin family of NAD+-dependent deacetylases; TCA—tricarboxylic acid cycle; LKB1—liver kinase B1; AMP—adenosine monophosphate; AMPK—AMP activated protein kinase; SOD—superoxide dismutase; CAT—chloramphenicol acetyltransferase; UCP2—uncoupling protein 2).

Proteins in OMM (mitofusin 1 and 2) [123], the inner mitochondrial membrane (IMM) (optic atrophy 1 (OPA1)) [124], and mitochondrial fission proteins (dynamin-related protein1 (DRP1)) [125] and fission1 [122] are stated to be damaged by ROS [122]. OPA1 was suggested to take part in mitochondrial respiratory efficiency [126], mitochondrial fragmentation and apoptosis [127], and its expression was found to be decreased in HFrEF patients [119]. It was discovered that the mitochondrial mitophagy marker BNIP3 takes part in promoting mitochondrial fragmentation by binding to OPA1, leading to OPA1 inhibition [127]. Additionally, BNIP3 inhibition increases DRP1 phosphorylation leading to its cytoplasmic translocation [117]. DRP1 was also found to be increased in HFrEF patients [119]. It is worth paying attention to mitochondrial dynamic proteins (MDPs) that are regulated by some signaling pathways involving proteasome-dependent degradation and transcription and, therefore, modulating mitochondrial function [128]. It was shown that MAPK phosphorylates MFN2, leading to its degradation, and is therefore important in myocardial remodeling induced by mitochondrial related apoptosis [129,130] (Figure 4).

Troponin I, phospholamban [129] and DRP1 [131] have been shown to be target proteins in PKA-related initiation of remodeling and progression to HF.

Calcium uptake in mitochondria is managed by the voltage-dependent anion channel (VDAC1) at the OMM [132], and is regulated by the mitochondrial calcium uniporter (MCU) at the IMM [133]. Calcium efflux is managed by the sodium/calcium/lithium exchanger at the IMM through intermembranous space, and then through VDAC1 into the cytosol [134]. Mitochondrial matrix calcium overload has been suggested as a major cause of mitochondrial dysfunction and decrease in oxidative capacity in patients with CHF [134], but decrease in MCU expression in HFrEF has also been suggested [135]. Therefore, clarification is needed on how activation of signaling pathways affects calcium flux through the OMM and IMM.

Decrease in oxidative capacity was found to be linked to decreasing expression of ETC complexes, or post-translation modifications of mitochondrial proteins (mainly acetylation) [117,119,136]. Cytochrome C oxidase activity was shown to be reduced along with reduction of expression of ETC complexes I and IV [117]. Decrease in ETC complex expression [119] and increase in protein acetylation is present in HFrEF patients [136]. Acetylation of ETC complexes, fatty acid beta-oxidation and tricarbonic acid (TCA) cycle proteins lead to inhibition of its activity [136] (Figure 4). It is speculated that the reason behind mitochondrial protein hyperacetylation is related to reduced protein deacetylation by SIRT3 and SIRT5, and excess of acyl-CoA [137]. Both SIRT3 and SIRT5 were found to be downregulated in a rat HFrEF phenotype, but not in a moderate cardiac remodeling setting [122].

Elevated cytoplasmic Na^+^, abnormal mitochondrial Ca^2+^ regulation and impaired energy metabolism are additional causes of diminished mitochondrial function in cardiomyocytes. These, in turn, downgrade mitochondrial energy supply and increase mitochondrial ROS release. Increased cytoplasmic Na^+^ impels mitochondrial Ca^2+^ depletion, mediated by mitochondrial Na^+^/Ca^2+^ exchanger activity. Therefore, qualitatively different patterns of ROS emission across a similar range of Ca^2+^ concentrations are produced [138].

A positive correlation between serum levels of reactive oxidative metabolites (DROM) and high-sensitivity C-reactive protein (hs-CRP) was shown [139], suggesting interfaces between oxidative stress and inflammation in HFrEF [140].

Post-ischemic condition, hemorrhage, severe trauma or toxic necrosis are the cause of sterile inflammation in HFrEF patients. As a result, endogenous stimuli trigger sterile inflammation by activating receptors, such as cluster of differentiation 36A, initializing the pathogen recognition receptor (PRR), resulting in tissue injury and intracellular cytokine release [141]. Subsequently, PRR triggers type I interferon (IFN), mitogen-activated kinase (MAPK) and nuclear factor kappa-B (NFκB), as a result, increasing pro-inflammatory chemokine and cytokine levels [142]. Therefore, granulopoiesis is induced by hematopoietic stem cells (HSC) that upregulate the production of neutrophils and monocytes [143]. Released neutrophils are conveyed through the blood into the heart where they phagocytose damaged cells. After infiltrating the heart, monocytes produce growth factors (IL-10, TGFβ) and cytokines to reduce inflammatory triggering and promote endothelial and smooth muscle cells to initiate scar formation [144]. This pathological event sequence causes cardiac fibroblasts to migrate and proliferate in the injury site where they are transformed into myofibroblasts. Stress fibers are produced by myofibroblasts, which cause interference and propagation of electrical signaling, as well as secretion of profibrotic signaling factors TNF-α, TGF-β and angiotensin II (Ang II). Together, these factors can induce and modify cardiomyocyte hypertrophy [145]. Moreover, myofibroblasts also line the extracellular matrix, resulting in interstitial and perivascular fibrosis that stiffens the myocardium, inducing collagenous scar formation [145]. Additionally, TNF-α triggers apoptosis in cardiomyocytes through death receptors [146].

Taken together, mitochondrial damage, including enzymes involved in metabolic pathways (such as fatty acid oxidation and the TCA cycle), OMM and IMM proteins, ETC protein acetylation and Ca^2+^ channels were found to be more related to ROS-induced cardiomyocyte damage and CHF progression in HFrEF patients. Mitochondrial-dysfunction-causing pathways can act simultaneously or subsequently contributing to HFrEF worsening. The possibilities for mitochondrial function improvement will be discussed in the treatment options section.

### 4.2. ROS in HFpEF

HF is defined as impaired LV myocardial contractility, diminished right ventricle (RV) function and decreased left atrium (LA) volumetric and contractile function. Energetic imbalances, interstitial fibrosis, cardiomyocyte hypertrophy, and oxidative stress derived from mitochondrial dysfunction and endothelial dysfunction, are always present in the setting of HFpEF [4,147].

Pro-inflammatory status affects multiple organ systems and contributes to generalized microvascular inflammation with diminished cyclic guanosine monophosphate (cGMP) and nitric oxide (NO) bioavailability, and reduced protein kinase G (PKG) activity. In turn, lessened PKG activity contributes greatly to cardiomyocyte hypertrophy and increased resting tension because of impaired connectin (a protein responsible for elasticity of the muscle) phosphorylation [141]. In addition, increased peroxynitrite concentrations, together with scarce NO availability, facilitate fibroblast proliferation through epidermal growth factor, platelet-derived growth factor, phosphatidylinositol 3-kinase and janus kinase pathways. These processes in combination cause stiffened cardiomyocytes and increased collagen deposits, which eventually lead to diastolic dysfunction due to elevated LV pressures in HFpEF [148,149,150]. Matrix metallopeptidase 9 (MMP9), a tissue inhibitor of MMP1, is also increased in HFpEF [151]. In addition, measured cardiomyocyte length and width were significantly larger in an HFpEF patient group, when compared with an HFrEF patient group [152].

**Figure 5:**
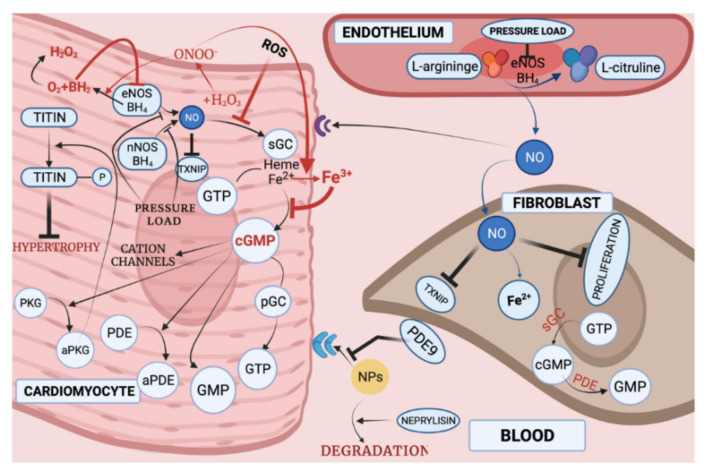
NO–cGMP-PKG pathway in HFpEF development (created with BioRender.com on 9 February 2022). NO, produced in endothelium by eNOS in normal conditions, protects fibroblasts and cardiomyocytes from harmful proliferation. BH4 (hydrobiopterin-4) is required for eNOS action. NO acts via stimulation of cardiac sGC receptors (leading to cGMP synthesis). cGMP regulates phosphodiesterases (PDEs) and cGMP-dependent protein kinases (PKG). NO inhibits TXNIP, resulting in inhibition of apoptosis, however ROS inhibit this action. Oxidative stress shifts sGC towards an oxidized heme-free form which is unresponsive to endogenous and exogenous NO. Titin hypophosphorylation leads to hypertrophy of cardiomyocytes. Increased peroxynitrite concentrations, together with scarce NO availability, induce fibroblast proliferation. Due to uncoupled eNOS, superoxide production increases. In turn, low levels of NO react with superoxide to generate peroxynitrite. Peroxynitrite: (1) oxidizes BH4 to BH2 (BH2 inhibits eNOS), and (2) oxidizes Fe^2+^ to Fe^3+^ (Fe^3+^ inhibits cGMP production from GTP). Therefore, cGMP cannot activate PKG to phosphorylate titin, whereas titin phosphate prevents cardiomyocyte hypertrophy. For this reason, eNOS inhibition results in both fibroblast and cardiomyocyte proliferation. Neprylisin catalyzes NPs degradation, and PDE9 inhibits NPs. NPs acts through receptors in cardiomyocytes to modulate proliferation of cardiomyocytes. NO—nitric oxide, eNOS—endothelial nitric oxide synthase, BH4—dihydrobiopterin-4, NPs—natriuretic peptide, PKG—protein kinase G, a—activated, sGC—soluble guanylyl cyclase, pGC—particulate guanylyl cyclase, PDE-GMP—regulated phosphodiesterase, ROS—reactive oxygen species, titin-P—phosphorylated titin, GTP—guanosine triphosphate, TXNIP—thioredoxin-interacting protein.

Comorbidities play an important role in the HFpEF setting. Diabetes mellitus, obesity, chronic kidney disease, hypertension and anemia generate a systemic inflammatory setting. For example, in obese HFpEF patients, macrophages in adipose tissue promote secretion of proinflammatory cytokines and, in CHF patients with anemia, low hemoglobin concentration fosters oxidative stress, caused by immune response to iron deficiency [153,154,155]. A systemic inflammatory state, induced by comorbidities, causes elevated levels of interleukin-6 (IL-6), tumor necrosis factor α (TNF-α), soluble ST2 (a receptor that inhibits cardioprotective impact of IL-33), and pentraxin 3 (a complement activator and autoimmunity control agent) [151,156,157]. In turn, pro-inflammatory cytokines induce ROS production in the endothelium through NOX [158], causing oxidative and nitrosative stress in the myocardium of HFpEF patients [159,160]. Obesity-induced inflammatory cytokines activate ROS production through NOX activation [161]. Decrease in NO has been shown to be highly important in HFpEF development through ROS [141] (Figure 5).

Due to uncoupled eNOS, superoxide production increases. In turn, low levels of NO react with superoxide to generate peroxynitrite, leading to nitration of tyrosine residues and formation of nitrotyrosine [162]. NO seems also to be responsible for early LV relaxation and reduction of end-diastolic stiffness [163]. In addition, NO acts by stimulating cardiac soluble guanylate cyclase (sGC) receptors, leading to cGMP synthesis [164]. Additionally, brain natriuretic peptide (BNP) stimulates particulate guanylate cyclase (pGC) [165]. cGMP is a secondary messenger, operating cGMP-gated cation channels, through cGMP regulated phosphodiesterases (PDEs) and cGMP-dependent protein kinases (PKG) [166]. In HFpEF, NO bioavailability is low due to inflammation and oxidative stress. cGMP concentration and PKG activity reduction was also observed in an HFpEF model. Moreover, oxidative stress shifts sGC towards an oxidized, heme-free form which is unresponsive to endogenous and exogenous NO [167]. Titin hypophosphorylation associated with stiffened cardiomyocytes was also observed in animal models [168,169]. Therefore, ROS are involved in the NO-sGC-cGMP-PKG pathway associated with titin hypophosphorylation and myocardial diastolic stiffness in HFpEF (Figure 5).

Structural vascular abnormalities are another important component in HFpEF pathogenesis. Stiffened arteries contribute greatly to increased pulse pressure and mean arterial and systolic blood pressures [170]. Mellisa A. Lyle et al. investigated contractile protein expression in HFpEF, HFrEF and control group patients and found that the HFpEF group had decreased myosin phosphatase target subunit 1-protein (responsible for NO mediated vasodilation) concentration. The authors, therefore, speculated that this was a possible reason why NO, cGMP or PKG signaling-pathway-targeted pharmacotherapies result in poor clinical benefits [171].

Inducible nitric oxide synthase (iNOS) upregulation is known to be an important factor in the development of HFpEF [172]. iNOS-related nitrosative stress increases s-nitrosylation of inositol-requiring protein 1α (IRE1 α) and decreases transcription factors involved in cellular stress response-spliced X-box-binding protein1 (XBP1s) levels [173]. It should also be mentioned that overexpression of XBP1s in cardiomyocytes weakened the HFpEF cardiac phenotype. It seems that iNOS-mediated nitrosylation of IRE1 α interferes with the XBP1 connection, which is required for the stress response [173]. Therefore, iNOS inhibition can be considered as a therapeutic strategy in the HFpEF setting.

Mitochondria play an important role in CHF, but are less understood in HFpEF pathophysiology [174]. Oxidative metabolism in mitochondria shifts towards a compensatory response through increased glycolysis; however, hypertensive HFpEF models do not seem to have correlation between increased glycolysis and, as a result, increased pyruvate oxidation rates, although it results in significant increase in proton production [175,176]. The same process was shown in an HFpEF-induced rat model, which displayed reduced pyruvate dehydrogenase activity, diminished glucose oxidation and increased pyruvate dehydrogenase kinase (PDK4) expression [177]. In obese models, mitochondrial ETC is affected through peak oxidative phosphorylation and NADH-associated respiration. NADH-associated dysfunction can be explained by diminished NADH-linked mitochondrial respiration due to calcium overload [178,179]. Declining energy reserves in the myocardium eventually lead to systolic dysfunction, even if it is “hidden” under a preserved ejection fraction [180].

Phosphodiesterase (PDE) 5 and 9 were shown to be upregulated in hypertrophy and CHF. In addition, PDE9-mediated natriuretic peptide (NPs) regulation in cardiomyocytes was more efficient than with NO-stimulated cGMP regulation [181]. PDE expression in the myocardium of HFpEF was increased and both PDE9 and PDE5 were involved in regulating cGMP-PKG activity [181]. Therefore, PDE9 and PDE5 could be potential treatment targets in patients with HFpEF.

Taken together, the NO-sGC-cGMP-PKG pathway appears to be the most investigated and important one in HFpEF pathogenesis. This pathway involves overproduction of hydrogen peroxide then initiates titin phosphorylation, leading to cardiomyocyte hypertrophy. Additionally, eNOS inhibition results in fibroblast proliferation. PDE is also involved in the cGMP induced pathway in HFpEF pathogenesis. The NO-sGC pathway has been investigated as a potential treatment target and will be discussed in the treatment strategy section of this article.

## 5. ROS-Induced Pathways as a Treatment Target in HFrEF and HFpEF

There are two interactive elements, related to other factors, that are implicit in the pathogenesis of CHF: inflammation and oxidative stress [182]. Some groups of compounds were investigated for their effect in oxidative stress-induced myocardial damage/remodeling reduction in CHF development including: (1) adenosine monophosphate-activated protein kinase (AMPK) activators, (2) renin-angiotensin system inhibitors (RAAS inhibitors), (3) inhibitors of ROS-producing enzymes, (4) antioxidants, (5) MAO inhibitors, (6) medications that improve mitochondrial function, and (7) substances that increase cGMP-PKG signaling. Each of these groups will be discussed below.

### 5.1. AMPK Activators

AMPK is an enzyme that has a pleiotropic cardioprotective impact and is important in the progression of CHF [84]. There are two AMPK isoforms: α1 and α2, and AMPK α2 seems to dominate in cardiomyocytes [183]. In normal conditions low amounts of ROS activate AMPK through different pathways [184], leading to antioxidant enzyme SOD, CAT and UCP2 gene expression (Figure 4), activation of pathways that produce adenosine triphosphate (ATP), suppression of apoptosis, inhibition of NOX expression and, by these pathways, protection from cardiac hypertrophy [146]. According to data presented above in the section on ROS in HFrEF, it appears that AMPK pathway action could be more feasible for HFrEF. Some drugs, such as metformin, statins, trimetazidine and resveratrol, were reported to have effects on AMPK activation and may prove to be beneficial in the clinical setting for ROS reduction and HF progression dampening [146]. For example, atorvastatin is suggested to activate the eNOS signaling pathway via AMPK, which, in turn controls NO bioavailability, maintains cardiovascular homeostasis and activates AMPK by altering the AMP/ATP ratio or increasing ROS-dependent PKC activity [146]. This process has been shown to attenuate heart dysfunction, fibrosis, and hypertrophy in a post-MI rat model [185]. Trimetazidine activates AMPK by influencing ATP levels in cardiomyocytes, thus improving heart function, New York Heart Association (NYHA) functional class, exercise tolerance and patient’s quality of life [186]. Resveratrol is stated to be able to inhibit cardiomyocyte hypertrophy through the AMPK-dependent pathway via two mechanisms: (1) at a high concentration (50–100 µM), it can activate AMPK by increasing the AMP/ATP ratio [187]; (2) the SIRT1-LKB1 (one of AMPK upstream activators) pathway [188]. Resveratrol in animal models decreases oxidative stress [189], but clinically it has not yet been thoroughly investigated [187,188]. AMPK not only improves energy supply to increase heart function, but also improves heart function by mediating various intracellular physiological functions, delaying myocardial fibrosis, and reducing heart damage in animal models. The benefits of these compounds in clinical studies with CHF patients appear to be worth exploring.

### 5.2. Renin-Angiotensin System Inhibitors

Angiotensin II (ANG II) is stated to promote excess accumulation of collagen [190] and is involved in ROS production in cardiomyocytes, leading to myocardial remodeling and chronic HF (Figure 4). Cardiomyocyte hypertrophy (induced by ANG II), therefore, could be inhibited by AT1R inhibition. Several potential drugs (e.g., valsartan, candesartan and kaempherol), affecting chronic HF pathogenesis through the ANG II pathway, have been investigated.

Treatment with valsartan reduced mRNA expression levels of NOX2 and NOX4, as well as the myocardial protein expression levels of NOX2 and NOX4, in rats with doxorubicin-induced myocardial injury [64]. The authors noted that ANG II increased the protein expression levels of NOX2 and NOX3 and the production of ROS, and that protein expression levels of ERK, JNK and P38, which lie downstream of MAPK, were increased as well. It was emphasized that pre-treatment with valsartan reduced the expression of AT1R, NOX2, NIX4 and ROS, therefore, activity of the MAPK signaling pathway was decreased [64]. Clinical studies have confirmed the valsartan effect on oxidative stress reduction through NOX2 in the human myocardium [191] (Table 1).

Neprilizine is an endopeptidase that cleaves the natriuretic peptides (NPs), bradykinin and adrenomedullin [195]. Inhibition of neprilysin is a main act due to an enhanced effect on biologically active NP. This inhibition increases the plasma concentrations of other vasoactive peptides, including vasodilators, such as adrenomedullin (a peptide associated with the calcitonin gene), bradykinin, and vasoconstrictor peptides, including endothelin-1 and angiotensin I and II [195]. Therefore, the AT1R inhibitor valsartan was added to neprilizine (sacubitril). It was discovered that sacubitril/valsartan (LCZ696—combination 1:1 of valsartan and sacubitril) reduced the risk of hospitalization for cardiac failure or death from cardiovascular disease in patients with HFrEF [196]. LCZ696 was also found to reduce plasma N-terminal pro b-type natriuretic peptide (NT-proBNP) concentration in HFpEF and to reduce the risk of death and hospitalization in HFrEF with EF ≤ 40% [192]; however, outcomes for HFpEF are not yet established. Therefore, LCZ696 provides a greater protection of target organs than AT1R therapy alone, including cardiovascular protection. This drug is superior in targeting the renin-angiotensin-aldosterone system (RAAS) in patients with HFrEF who can tolerate AT1R inhibitors, with a better safety and efficacy profile [190,192,197]. LCZ696 was well tolerated in a Phase II large HFrEF population [192], produced lower levels of NTproBNP (NCT01920711) and improved NYHA functional class [198,199].

Researchers recently investigated the usefulness of the ATR1 locator candesartan in HFpEF treatment and discovered that candesartan improved outcomes to a similar degree as for HFrEF patients [194].

Current guidelines strongly recommend neurohormonal antagonist treatment for HFrEF [200]. Despite HFpEF patients representing a majority of those with chronic HF in the general population, there are no recommendations for HFpEF treatment with sacubitril/valsartan [201].

Kaempherol (KFP, 3,4’,5,7-tetrahydroxyflavone) is a flavonoid, found abundantly in plant foods [202], and prevents oxidative stress [203]. KFP was suggested to inhibit cardiac remodeling through deactivation of mitogen-activated protein kinases (MAPKs) [204]. The activation of MAPK is known to promote fibrosis (Figure 3). Additionally, treatment aimed at cardiac fibroblasts with KFP resulted in decreased expression of pro-inflammatory cytokines [204], supplementing the cardioprotective effect. Further clinical studies are needed to determine the suitability of this medication in patients.

### 5.3. Inhibitors of ROS-Producing Enzymes

The mitochondrial and cytosol enzymes NOX, NOS and XOR were discussed as primarily ROS-produced enzymes in cardiomyocytes (Section 3.1 and Section 3.2). ETC proteins were suggested as ROS sources as well. One of the compounds related to mitochondrial ETC is Mito-Q—a combination of the triphenylphosphonium cation (TPP) and Q_10_. This remedy demonstrated promising antioxidative effects in several human studies [205,206,207,208]. Mito-Q is Q_10_ coupled to lipophilic TPP+ and it accumulates on the IMM [205]. Q_10_, when reduced to ubiquinol by the ETC, acts as an antioxidant, preventing mitochondrial oxidative damage [206] and resulting in heart hypertrophy reduction in an HF rat model [208]. MitoQ is stored in mitochondria in vivo and is a part of the redox system, together with the reduced hydroquinone MitoQuinol form. The pivotal aim of MitoQ is to protect and prevent cellular damage, triggered by mitochondrial ROS overproduction and oxidative stress [209,210]. It is important to mention that MitoQ is bound to the mitochondrial IMM, mostly in the hydrophobic membrane core, which is determined by the membrane potential, while the respiratory chain complex II is continuously processed into ubiquinol [211]. The active part of MitoQ is ubiquinone (coenzyme Q_10_) [212]. MitoQ was shown to protect against oxidative damage in animal models with HF by reducing hydrogen peroxide formation [207]. Further clinical studies are needed to confirm the analogous effect in larger humans.

NOX-, XOR- and NOS-induced pathway inhibition may be one of the ways to reduce ROS damage in the heart. In small clinical studies, it has been shown that myocardial O_2_ consumption is lowered and mechanical efficiency of the LV is improved by XOR inhibition [56] due to increased ATP flux through creatine kinase (CK) [213]. XOR inhibitors are known to improve LVEF [214], endothelial function [215], and to decrease BNP levels [216] in HF patients. However, larger studies did not show the same amount of benefit in an HFrEF patient group [217,218,219].

Inhibition of eNOS and combined treatment with BH4 also reduced ROS production in animal models with HF [63]. Overexpression of the enzyme catalyzing BH4 biosynthesis (GTP-cyclohydrolase 1) or oral consumption of BH4 shielded from harmful Ca^2+^ pathways and contractile dysfunction in isolated cardiomyocytes in vivo mice through nNOS action [220]. However, few clinical studies have investigated the benefits of BH4 usage and the results of these were rather poor.

Taken together, XOR inhibitors have been shown not to be effective in chronic HF patient treatment. Management of NOS activity is still at the research stages. We have not found any studies that investigated the benefits of NOX inhibition or Mito-Q effect in CHF patients.

### 5.4. Antioxidants

Administration of antioxidants (AOx) has been expected to be a simple and effective way to reduce oxidative harm in the myocardium. Vitamin-antioxidants, Mito-TENPO, enzyme-scavengers of ROS and elamipretide were investigated (Table 2).

Some small studies evaluated vitamin-antioxidants combinations in HFpEF [216] and HFrEF [221] and discovered improvement in peripheral vascular function and decrease in oxidative stress (Table 2). In the first study, reactive hyperemia (RH), a measure of microvascular function, did not change after OC (combination of α-lipoic acid, vitamin C and vitamin E) administration. Improvement in flow-mediated dilation was accompanied by significant increase in plasma nitrite and decrease in CRP, but additional biomarkers of oxidative stress, plasma concentrations of free radicals and antioxidant capacity were not altered by AOx. These findings confirm the efficacy of an over-the-counter OC combination in achieving systemic anti-inflammatory effects and improving peripheral vascular function, regardless of changes in global markers of oxidative stress in HFpEF, providing new insight into the potential therapeutic effect of AOx [216]. The second study of HFrEF patients was characterized by macrovascular endothelial dysfunction, which may be due, at least in part, to a change in redox balance, leading to increased oxidative stress and decreased endogenous AOx protection. The results of this small study showed that chronic AOx administration is a simple way to improve macrovascular function, reduce oxidative stress, and increase AOx capacity in patients with HFrEF [221] (Table 2). However, larger sample studies are needed to properly investigate the beneficial effects of this compound.

Mito-TENPO is a mitochondria-targeted chemical with superoxide-scavenging properties [208]. MitoTENPO was given to prevent and reverse HF [222] and improved LV contraction [223] in a mouse model. Despite these promising results, we did not find any studies performed with CHF patients.

One more area of AOx application in chronic HF therapy is enhancing ROS scavenging capacity through GSH [224], SOD [225] and catalase [226]. Different chemicals were investigated to increase SIRT3 activity as well [204]. Studies in these fields demonstrated beneficial results, but further investigations are still needed.

Despite increasing interest in oxidative stress management possibilities in CHF patients, most antioxidant therapies are not successful [6,227].

### 5.5. MAO Inhibitors

MAO are enzymes located on the OMM. They catalyze deamination of biogenic amines and neurotransmitters [102]. The mechanisms of MAO toxicity have been commonly associated with excessive H_2_O_2_ production, due to MAO appearing to be one of the major ROS sources within the mitochondria [100]. Studies in animal models suggested that activation of MAO-A/B plays a crucial role in progression from cardiac hypertrophy to cardiac failure, establishing a clear association between MAO-induced ROS production and mitochondrial dysfunction. However, MAO is suggested as a promising new therapeutic target in chronic diseases [228,229,230,231,232,233,234,235]. Despite the clinical relevance of these findings, and the possible indications for MAO inhibitors in the treatment of chronic HF, little is known about the activity of MAO in HF patients and its association with redox imbalance [236].

### 5.6. Mitochondrial Function Improvement

Mitochondrial function depends mainly on ETC and membrane integrity. Coenzyme Q (CoQ_10_) is one of the ETC components [237]. Currently, only one clinical study is being conducted to address this aspect of the pathophysiology of HFpEF. A study on CoQ_10_ in diastolic heart failure patients (NCT03133793) is investigating the efficacy of ubiquinol, a reduced form of CoQ_10_, which acts to reduce the severity of HFpEF symptoms and improve cardiac function [238].

CoQ_10_ is an essential cofactor of the ETC from complexes I and II to complex III. It maintains mitochondrial membrane potential, supports ATP synthesis and inhibits ROS generation [239,240]. Plasma levels of CoQ_10_ are decreased in patients with chronic HF, and correspond to the severity of a disease. Doses of CoQ_10_ have been reported to increase the incidence of adverse reactions at doses above 1200 mg/day, with doses of 22 to 400 mg/day being considered safe [241]. An analysis of small studies suggested that CoQ_10_ can improve LVEF in HFrEF [242] and can reduce cardiovascular mortality by 50% [243]. However, it was not sufficiently powerful due to poor prognostic effect [237] for it to be recommended in guidelines [5]. CoQ_10_ is also needed for eNOS management [235]. CoQ_10_ benefits in HFrEF patients are well discussed by A. Sharma and co-authors [237]. It remains unclear if prescribing of CoQ_10_ is useful due to mitochondrial function improvement, or due to its involvement in eNOS action. The utility of CoQ_10_ for HFpEF patients requires further investigation.

Elamipretide is a compound that accumulates in the IMM by binding to cardiolipin [244], a phospholipid, required for proper ETC function and other IMM proteins [244,245]. Cardiolipin can be oxidized by elevated ROS [246] and disturbs interaction of ETC complexes, leading to O^2−^ increase and apoptosis initiation [247]. Elamipretide improved mitochondrial function in a dog model [248] and isolated cardiomyocytes [249,250]. Despite favorable effects in animal models and isolated cardiomyocytes, the clinical effects in humans with HF are rather modest (Table 3).

### 5.7. Chemicals Increasing cGMP-PKG Signaling

Constantijn Franssen, with co-authors, reviewed studies conducted before the year 2014 regarding medications that increased cGMP-PKG signaling [253]. The medicines reviewed were nitroxyl (HNO), enalapril, LCZ696 and sGC activators (e.g., cinaciguat, riociguat, vericiguat). HNO was shown to increase cGMP and to suppress NOX, resulting in an anti-hypertrophic effect in rat cardiomyocytes [254] and HFrEF patients [255], but clinical studies of the HNO effect in HFpEF were absent. LCZ696 was discussed in the chapter, *“Renin-angiotensin system inhibitors”.* Cinaciguat did not show any effect on cardiac index [256]. Riociguat improved symptoms and NT-proBNP levels [257]. Vericiguat was evaluated in a Phase II trial study in HFrEF and HFpEF [258].

Knowledge about medications that increase cGMP-PKG signaling has grown in the last decade. Sodium-glucose cotransporter 2 inhibitor (SLGT2), soluble guanylate cyclase (sGC) activators, PDE inhibitors, NO donors, and the vasodilator hydralazine were investigated.

Empagliflozin (sodium-glucose cotransporter 2 inhibitor) and sGC activator were suggested to have antioxidant and anti-inflammatory features in the myocardium of HF rats and HFpEF patients [259]. The results revealed that empagliflozin reduced cardiovascular mortality, all-cause mortality, and the number of hospitalizations for HFrEF. Moreover, both empagliflozin and sGC activator improved cardiomyocyte function by enhancing the phosphorylation of titin and other myofilament proteins, presumably due to improved signaling pathways, such as the nitric oxide (NO)/soluble guanylyl cyclase (sGC)/cGMP-dependent protein kinase (PKG) signaling pathway (NO-sGC-cGMP-PKG pathway) and the CaMKII-mediated hypertrophic pathway, PKC, ERK2, in addition to the PKA pathway [259] (Figure 5).

The PDE5 inhibitor sildenafil can inhibit guanosine 3′,5′-cyclic monophosphate (cGMP) breakdown, improve cardiac relaxation and LV remodeling [260]. The catalytic site of PDE5 generally degrades cGMP, and sildenafil potentiates the endogenous increase in cGMP by inhibiting its degradation [261]. Sildenfil reduced pulmonary vascular resistance and right heart pressure in patients with HFrEF, who had secondary pulmonary hypertension, and long-term treatment improved exercise tolerance, functional capacity, LV diastolic function and cardiac geometry [262,263,264]. In a large, long-term (24 weeks) trial of sildenafil (RELAX), PDE5 inhibitor did not improve LV diastolic function and did not reduce hypertrophy and pulmonary pressures. In this study, sildenafil did not increase plasma cGMP concentrations, therefore, exercise capacity and clinical status did not improve [265].

NO donors were also investigated for their capacity to improve heart function. Some of these donors, such as Angeli’s salt [266] and Piloty’s acid [267], appeared to be unstable. In turn, pure NO donors, such as the congener of Piloty’s acid, CXL-1020 [255], and the pro-drug of CXL-1020, cimlanod (BMS-986231), were generated [268]. The HNO donor BMS-986231 in animal models improved myocardial contractility and relaxation without increasing heart rate or oxygen consumption [269]. One study was performed with HFrEF, in which patients received intravenous infusions (i.v.) of BMS-986231 at various doses, and information about the safety and tolerability of medicine was provided [270]. However, the poor solubility of BMS-986231 limited its clinical use as an i.v. agent, and its oral bioavailability is still being investigated [268].

Major studies were performed with the protonated form of NO—nitroxyl (HNO). The action of HNO is preserved during oxidative stress because HNO does not react with superoxides [271], and undergoes moderate oxidative reactions through the formation of hydroxyl radicals [272]. HNO inhibits mitochondrial respiration by inhibiting complexes I and II, most likely by modifying specific cysteine residues in ETC proteins [273]. HNO increased cGMP levels and had NADPH oxidase (NOX2) inhibitory and antihypertrophic effects in rat cardiomyocytes [255]. HNO improved myocardial function due to direct positive lusitropic and inotropic effects, independent of cyclic adenosine monophosphate (cAMP), and due to combined venous and arterial dilation [274,275,276,277,278]. In addition, HNO modifies sarcomeric proteins to increase their Ca^2+^ sensitivity resulting in systolic force generation [270]. HNO also causes vasodilatation through endothelial soluble guanylate cyclase [266,279]. A recent study showed that HNO reduced left and right ventricle filling pressure and systemic vascular resistance in both animal and CHF patient models [255]. It was concluded that nitroxyl was well tolerated, reduced diastolic filling pressure and systemic vascular resistance, and raised cardiac output and stroke volume with unaltered heart rate [255]. Taken together, the novel cardio-protective properties of HNO show the therapeutic potential of HNO donors, particularly in situations where NO signaling is impaired (in HFpEF), but more detailed studies are required.

The vasodilator hydralazine has a beneficial effect on the balance between NO and O_2_, which is disturbed in patients with HF [280]. Clinical treatment with nitrates resulted in eventual tolerance to its vascular and hemodynamic effects, mainly due to endothelial dysfunction [281]. However, in combination with hydralazine, nitrate tolerance was avoided due to hydralazine inhibition on nitroglycerin-induced vascular O_2_, and peroxynitrite (ONOO^−^) formation in vitro [282] and in vivo [283]. Thus, the antioxidant effect of hydralazine and the prevention of the development of its tolerance in response to isosorbide dinitrate (ISDN) [284] may at least partially explain why this combination improves morbidity and mortality in patients with chronic congestive HF [285]. The African-American Heart Failure (A-HeFT) trial demonstrated that ISDN and hydralazine combination has a large effect on survival in patients with HFrEF [286].

Recently, new classes of drugs that increase cGMP production by targeting guanylate cyclase at the NO receptor (sGC) have been discovered. These were designed in order to generate cGMO independently of NO and to target signaling cascades in the cardiovascular system [287]. Enzymes with a unique mode of action activate the oxidized, heme-free form of sGC, which does not react with NO. The oxidation or absence of the heme moiety increases the effect of cinaciguat on the sGC, causing a significant cGMP increase [288]. These compounds are called sGC stimulators and sGC activators. They differ in that sGC stimulators are targeted to bind to the regulatory domain and trigger cGMP production by binding the heme-containing non-oxidized form of the sGC regulatory domain [289].

In recent years, the soluble stimulant sGC vericiguate has attracted the attention of the medical community following reports of reduced clinical outcomes in patients with chronic heart failure. The NO-sGC-cGMP pathway is mediated by a different mechanism that complements current drug therapy for cardiovascular disease. cGMP deficiency is a characteristic trait of both HFrEF and HFpEF [258]. Vericiguate acts synergistically with endogenous NO [285,290], which is considered a nitroconstrictor that produces cGMP at low levels of NO (Figure 5). By increasing cGMP, vericiguate has also been shown to promote vascular relaxation and improve vascular tone regulation and myocardial dysfunction [285,291,292,293]. This would also attenuate left ventricular remodeling by inducing PKG-induced phosphorylation of titin after activation of PKG by cGMP [292] (Figure 5). sGC activators are well discussed by Chien Y.T. et all [294]. Vericiguat is currently in phase 3 clinical trials for HFrEF (BAY 1021189) and praliciguat is now in phase 2, in HFpEF (IW-1973, IWP-121) [294]. Drugs that alter cardiomyocyte homeostasis by increasing cGMP-PKG signaling in HF patients are summarized in Table 4.

To conclude, AMPK activators are suggested to improve heart function in animal models, and, with further research, have potential to be beneficial for patients with chronic HF. The AT1R and neprilysin inhibitor valsartan/sacubitril has been investigated in most detail of all Ang II inhibitors and was included in guidelines for HFrEF treatment. Kaempherol displayed cardioprotective effects in cell culture [203,204]; however, further clinical studies are needed in order to assess its suitability and beneficial effects for patients. The utility of antioxidants, mitochondrial-function-affecting drugs and MAO inhibitors have been poorly studied to date. Both the SLGT2 inhibitor, sGC activator empagliflozin and vericiguat produced gratifying treatment results in patients with HFpEF.

## 6. Conclusions

Mitochondrial damage, inflammation and enzyme-oxidants (NOX, XOR, NOS), as well as decreased activity of enzyme-antioxidants (GPX and PRX), can be assumed to be the main triggers for excess amounts of ROS in cardiomyocytes. AT1R is involved in NOX activation in both cardiomyocytes and fibroblasts. Enzyme-oxidants act through MAPK and NO synthesis inhibition pathways, leading to cardiomyocyte hypertrophy and interstitial fibrosis. Pro-inflammatory cytokines trigger ROS overproduction, leading to mitochondrial structural damage due to membrane protein, ion channel protein and protein acetylation in HFrEF mitochondria. Therefore, damaged mitochondria are suggested to be the main ROS source in HFrEF, while NO decrease, NO-sGC-cGMP signaling inhibition by ROS and enzymes iNOS, eNOS, PDE are understood to be the most important factors in HFpEF development.

The pathways involving both AMPK and MAPK protein kinases, AT1R and cGMP-PKG, are considered as treatment targets for halting chronic HF development. Therefore, some compound groups for oxidative stress-induced myocardial damage/remodeling reduction in HF development include: (1) activators of AMPK, (2) RAAS inhibitors, (3) inhibitors of ROS producing enzymes, (4) antioxidants, (5) MAO inhibitors, (6) medications that improve mitochondrial function, and (7) substances that increase cGMP-PKG signaling.

Achievements in reducing ROS-induced harmful pathways in chronic HF can be summarized as follows: AMPK activators are suggested to improve heart function in animal models, therefore, exploratory studies with patients afflicted with chronic HF could prove to be of great value. AT1R and the neprilysin inhibitor valsartan/sacubitril have been investigated in the most detail of all. Ang II inhibitors have yielded favorable results and been included in HFrEF treatment guidelines. Kaempherol displayed cardioprotective effects in animal models, but clinical studies are still needed to verify the suitability and treatment benefits in humans. XOR inhibitors and management of the NOS activity in chronic HF patient treatment are still in research stages. Further studies that investigate the benefits of NOX inhibition or Mito-Q effect in CHF patients are needed. The usefulness of antioxidants, mitochondrial-function-affecting drugs and MAO inhibitors are still poorly studied and understood. Both the SLGT2 inhibitor and the sGC activator empagliflozin and vericiguat displayed gratifying results in HFpEF treatment; however, their effects still require to be confirmed in randomized studies.

## 7. Perspectives

ROS were shown to be involved in both HFrEF and HFpEF pathogenesis through different pathways. Although treatment with unselective antioxidative treatment failed to demonstrate better outcomes in HFrEF and HFpEF patients, oxidative stress remains the focus of intensive research. It appears that selective antioxidant treatment ought to give more favorable results. Treatment regarding mitochondrial function improvement and cGMP-PKG signaling appear to need deeper investigation for both CHF patient groups. In HFrEF, patient usage of AMPK activators should be evaluated.

## Figures and Tables

**Table 1:** Medicines affecting human cardiomyocytes via renin-angiotensin system in HF patients.

Medicine	Patients	Appl., Dose and Duration	Results	Pathophysiological Mechanism	Reference
Valsartan	CHF, NYHA functional class II–IV, *n* = 83	6-week study, 80 or 160 mg bid	Produced hemodynamic and hormonal effects.	Blocks angiotensin AT1 receptor leading to NOX2 activity reduction.	[191]
Sacubitril/valsartan (LCZ696)	HFrEF (NYHA II–IV) and LVEF ≤ 40% *n* = 4822	5 years	Decreased levels of NT-proBNP or improved left atrial volumes.	Inhibits neprilysin/ ATR	[192]
Sacubitril/valsartan	HFrEF, *n* = 54	Twice a day 24/26, 49/51, 97/103 mg	Improved NYHA class, decreased NT-pro BNP concentration, reduced mortality.	Inhibits neprilysin/ ATR1	[193]
Candesartan	HFpEF *n* = 1958, HFrEF, *n* = 1959	2.9 years	Improved outcomes in both groups.	ATR1 inhibitor	[194]

**Table 2:** Antioxidants affecting human cardiomyocytes in chronic HF patients.

Medicine	Patients	Appl., Dose and Duration	Results	Pathophysiological Mechanism	Reference
OTC OCs	Patients with heart failure with preserved ejection fraction (HFpEF), *n* = 16.	600 mg of α-lipoic acid, 1000 mg of vitamin C, and 600 IU of vitamin E	Improved peripheral vascular function regardless of changes in global markers of oxidative stress in HFpEF.	Alterations in redox balance as a result of attenuated endogenous AOx capacity and/or elevated oxidative stress might be an underlying mechanism.	[216]
AOx	Patients with HFrEF, *n* = 14.	1 g of vitamin C, 600 IU of vitamin E, and 0 mg/day). 6 g α). -lipoic acid	Improved macrovascular function, reduced oxidative stress, and increased AOx capacity in patients with HFrEF	Changes redox balance; Increases oxidative stress; Decreases endogenous AOx protection.	[221]

**Table 3:** Medicines affecting human cardiomyocyte mitochondria in chronic HF patients. (LVEDV- LV end-diastolic volume, LVESV-LV-end-systolic volume).

Medicine	Patients	Appl., Dose and Duration	Results	Pathophysiological Mechanism	Reference
Elamipretide (SS-31)	HFrEF(EF ≤ 35%), *n* = 24 and placebo *n* = 12.	i.v., 4-h infusion 0.25 mg × kg^−1^ × h^−1^	↓LVESV, ↓LVEDV	By binding to cardiolipin, decreases ROS production.	[251]
Elamipretide (SS-31)	HFrEF(EF≤40%), *n* = 48 and placebo *n* = 23.	p.o., 4 mg or 40 mg once daily for 28 days.	Did not improve LVESV.	By binding to cardiolipin, decreases ROS production.	[252]
Coenzyme Q_10_	Moderate to severe HFrEF, *n* = 420.	p.o., 100 mg 3 times daily, 2 years.	Significantly improved NYHA class, CV events ↓by 50%.	Q10 is involved in eNOS regulation	[243]

**Table 4:** Medicines affecting human cardiomyocytes by increasing cGMP-PKG signaling in HF patients. (LV-left ventricle, LA-left atrium).

Medicine	Patients	Appl., Dose and Duration	Results	Pathophysiological Mechanism	Reference
Empagliflozin	HFpEF II–IV class (EF > 40%), *n* = 2997, placebo *n* = 2991.	10 mg once daily or placebo 36 months	Reduced cardiovascular death and hospitalization	SLGT2 inhibitor and sGC activator	[295]
Sildenafil	Stable outpatient individuals with HFpEF, *n* = 160.	24 weeks	Did not improve exercise capacity and clinical status.	Inhibits cGMP breakdown	[265]
Nitroxyl	HFpEF, *n* = 65.	23 months	HNO increased cGMP concentrations and had NOX inhibitory and antihypertrophic effects in rat cardiomyocytes.	NO donor.	[255]
Cymlanod	HFrEF, *n* = 45	5 h i.v infusion or placebo	Slightly reduced LV and LA volumes	NO donor	[296]
Hydralazine	HFrEF, NYHA class III–IV (EF ≤ 35% or < 45% with LVIDd > 2.9 cm/m), *n* = 1050.	18 months	Improved survival.	ROS scavenger; Inhibitor of O_2_^−^generation; normalizes endogenous rates of vascular O_2_^−^production [122].	[286]
Cinaciguat	HFrEF. *n* = 62	1 year	Did not significantly improve dyspnea or cardiac index.	Increases cGMP production by targeting guanylate cyclase at the NO receptor (sGC).	[256]
Vericiguat	HFrEF, (LVEF < 45%, history of decompensation within the last four weeks), *n* = 456	1.25 mg, 2.5 mg, 5 mg, or 10 mg for 12 weeks	Was well-tolerated and higher doses were associated with a greater reduction in NT-pro BNP level.	Triggers cGMP production by binding the heme-containing non-oxidized form of sGC regulatory domain.	[290]
	HFpEF ( LVEF > 45% and a history of decompensation within the last four weeks), *n* = 477	from 1.25 mg to 10 mg once daily for 12 weeks	Appeared to be well-tolerated and improved patients with HFpEF quality of life; however, had no significant impact on NT-proBNP level.		[297]
	HFpEF (LVEF > 45%), *n* = 789	15 mg or 10 mg daily	No significant changes were observed.		[298]
	HFpEF (NYHA II to IV) with an LVEF < 45%, history of decompensation over the last six months, elevated NT-proBNP or BNP), *n* = 5050	10 mg once daily for 10.8 months	Hospitalization for heart failure and death from cardiovascular causes were reduced compared to placebo.		[299]

## Data Availability

Not applicable.

## Abbrevations

ALDH2	aldehyde dehydrogenase
AMP	adenosine monophosphate
AMPK	adenosine monophosphate activated protein kinase
ANGII	angiotensin II
AO	antioxidant
AOx	vitamin C, vitamin E, α-lipoic acid (antioxidants)
AT1R	angiotensin II receptor
ATP	adenosine triphosphate
BH4	dihydrobiopterin-4
BNIP3	mitochondrial mitophagy marker
BNP	brain natriuretic peptide
Bax	bcl-2-like protein 4
CAT	chloramphenicol acetyltransferase
CHF–	chronic heart failure
CI	ETC complex I
CIII	ETC complex III
CK	creatine kinase
CRP–	C-reactive protein
Cluh	clustered mitochondria protein homolog
CoenzymeQ-10–	ubiquinone
DM–	diabetes mellitus
DROM	reactive oxidative metabolites
DRP1	dynamin related protein1
ERK	extracellular signal-regulated kinase
ERK1/2	extracellular signal-regulated kinase 1/2
ETC	electron transport chain
GTP	guanosine triphosphate
GMP	guanosine monophosphate
GPX	glutathione peroxidase
GSH	glutathione
H_2_O2	hydrogen peroxide
HF	heart failure
HFpEF	heart failure with preserved ejection fraction
HFrEF	heart failure with reduced ejection fraction
HNO	nytroxyl
HSC	hematopoietic stem cells
IDH	isocitrate dehydrogenase
IDH2	isocitrate dehydrogenase
IL-10	interleukin-10
IL-6	interleukin-6
IMM	inner mitochondrial membrane
IRE1α	inositol-requiring protein 1α
ISDN	isosorbide dinitrate
JNK-c	Jun N-terminal kinase
KFP	Kaempherol
LA	left atrium
LKB1	liver kinase B1
LPHs	lipid hydroperoxide
LV	left ventricle
LVEDV	left ventricle end-diastolic volume
LVESV	left ventricle end-systolic volume
LVH	left ventricular hypertrophy
LVID	left ventricular internal dimension
MAO	monoamine oxidase
MAO-A	monoamine oxidase A
MAPK	mitogen-activated protein kinase
MCU	mitochondrial calcium uniporter
MMPs	matrix metalloproteinase
NAPDH-	nicotinamide adenine dinucleotide phosphate
NFκB	nuclear factor kappa-B
NHE-1	Na^+^/Ca^2+^ exchanger
NO	nitric oxide
NO-2	plasma nitrite
NOS	nitric oxide synthase
NOX	nicotinamide adenine dinucleotide phosphate oxidase
NP	natriuretic peptide
NT-proBNP	N-terminal pro b-type natriuretic peptide
NYHA-	New York Heart Association
OMM	outer mitochondrial membrane
ONOO^−^	peroxynitrite
OPA1	optic atrophy 1
P38	a focal point of interactions of the serine/threonine kinases
PDE	phosphodiesterases
PKA	protein kinase A
PKB	protein kinase B
PKC	protein kinase C
PKG	protein kinase G
PKS	protein kinase S
PRR	pathogen recognition receptor
PRX	peroxiredoxin
RAAS	renin-angiotensin-aldosterone system
ROS	reactive oxygen species
Rac-1	GTP-binding protein
SGLT2	sodium-glucose cotransporter
SIRT	NAD+ dependent class III histone deacetylases
SOD	superoxide dismutase
TGFβ	tumor growth factor β
TNF-α	tumor necrosis factor-α
TPP	triphenylphosphonium cation
TXNIP	thioredoxin-interacting protein
UCP2	uncoupling protein 2
VDAC1	voltage-dependent anion channel 1
XBP1s	X-box-binding protein1
XO	xanthine oxidase
XOR	xanthine oxidoreductase
cAMP	cyclic adenosine monophosphate
cGMP	cyclic guanosine monophosphate
eNOS	endothelial nitric oxide synthase
hs-CRP	high-sensitivity C-reactive protein
iNOS	inducible nitric oxide synthase
nNOS	neuronal nitric oxide synthase
pGC	particulate guanylyl cyclase
sGC	soluble guanylate cyclase
β-OX	beta-oxidation
